# NEW TECHNIQUE FOR OBESITY SURGERY: INTERNAL GASTRIC PLICATION TECHNIQUE USING INTRAGASTRIC SINGLE-PORT (IGS-IGP) IN EXPERIMENTAL MODEL

**DOI:** 10.1590/0102-6720201700010017

**Published:** 2017

**Authors:** Verena MÜLLER, Panagiotis FIKATAS, Safak GÜL, Maximilian NOESSER, Kirs ten FUEHRER, Igor SAUER, Johann PRATSCHKE, Ricardo ZORRON

**Affiliations:** 1 Center for Innovative Surgery (ZIC), Center for Bariatric and Metabolic Surgery, Department of General, Visceral and Transplant Surgery, Campus Virchow Klinikum and Department of General, Visceral, Vascular and Thoracic Surgery, Campus Mitte, Charité-Universitätsmedizin, Berlin, Germany.

**Keywords:** Morbid obesity, Bariatric surgery, Laparoscopy, Endoscopic sleeve gastroplasty, Intragastric sleeve gastroplication

## Abstract

**Background:**

Bariatric surgery is currently the most effective method to ameliorate co-morbidities as consequence of morbidly obese patients with BMI over 35 kg/m^2^. Endoscopic techniques have been developed to treat patients with mild obesity and ameliorate comorbidities, but endoscopic skills are needed, beside the costs of the devices.

**Aim:**

To report a new technique for internal gastric plication using an intragastric single port device in an experimental swine model.

**Methods:**

Twenty experiments using fresh pig cadaver stomachs in a laparoscopic trainer were performed. The procedure was performed as follow in ten pigs: 1) volume measure; 2) insufflation of the stomach with CO2; 3) extroversion of the stomach through the simulator and installation of the single port device (Gelpoint Applied Mini) through a gastrotomy close to the pylorus; 4) performance of four intragastric handsewn 4-point sutures with Prolene 2-0, from the gastric fundus to the antrum; 5) after the performance, the residual volume was measured. Sleeve gastrectomy was also performed in further ten pigs and pre- and post-procedure gastric volume were measured.

**Results:**

The internal gastric plication technique was performed successfully in the ten swine experiments. The mean procedure time was 27±4 min. It produced a reduction of gastric volume of a mean of 51%, and sleeve gastrectomy, a mean of 90% in this swine model.

**Conclusion:**

The internal gastric plication technique using an intragastric single port device required few skills to perform, had low operative time and achieved good reduction (51%) of gastric volume in an in vitro experimental model.

## RESUMO

**Racional:**

A cirurgia bariátrica é atualmente o método mais efetivo para melhorar as co-morbidades decorrentes da obesidade mórbida com IMC acima de 35 kg/m^2^. Técnicas endoscópicas foram desenvolvidas para tratar pacientes com obesidade leve e melhorar as comorbidades, mas habilidades endoscópicas são necessárias, além dos custos.

**Objetivo:**

Relatar uma nova técnica para a plicatura gástrica interna utilizando um dispositivo intragástrico de portal único em modelo experimental de suínos.

**Métodos:**

Foram realizados 20 experimentos utilizando estômagos de cadáver de porco fresco em um instrutor laparoscópico. O procedimento foi realizado da seguinte forma em dez porcos: 1) medida de volume; 2) insuflação do estômago com CO2; 3) extroversão do estômago através do simulador e instalação do dispositivo de uma única via (Gelpoint Applied Mini) através de uma gastrotomia próxima ao piloro; 4) realização de quatro suturas de quatro pontos intra-gástricas com Prolene 2-0, desde o fundo gástrico até o antro; 5) medição do volume residual. A gastrectomia vertical foi também realizada em mais dez suínos e o volume gástrico pré e pós-procedimento foi medido.

**Resultados:**

A técnica de plicatura gástrica interna foi realizada com sucesso nos dez experimentos com suínos. O tempo médio do procedimento foi de 27±4 min. Produziu redução do volume gástrico em média de 51%, e a gastrectomia vertical em média de 90% neste modelo suíno.

**Conclusão:**

A técnica de plicatura gástrica interna, utilizando um dispositivo intragástrico de uma única via, exigiu poucas habilidades para ser realizada, teve baixo tempo operatório e obteve boa redução (51%) do volume gástrico em um modelo experimental in vitro.

## INTRODUCTION

The increase of obesity to one third of the world population^1^ calls for more than 370 million people who are currently suffering type 2 Diabetes Mellitus (T2DM)^2^metabolic surgery has been shown to be more effective in reducing mortality, improving hyperglycemia, hypertension and dyslipidemia in randomized clinical trials among patients with obesity and type 2 diabetes. However, surgery also has the risk for acute perioperative complications, long-term micronutrient deficiencies and psychological problems. Weighing these risks against the benefits of significant weight loss and improved glycemic control, metabolic surgery seems to be a promising treatment option for obesity-associated type 2 diabetes. However, current guidelines and treatment algorithms for the treatment of type 2 diabetes either ignore or underestimate the potential of metabolic surgery. In my opinion, metabolic surgery should be considered earlier in the treatment of type 2 diabetes and obesity and no longer be considered as the last therapeutic option for patients with obesity-associated type 2 diabetes.. Conservative medical treatment for obesity and/or T2DM does not seem to be successful. Multimodal therapies such as exercises, dietary change and cognitive therapy alone are little effective. Bariatric surgery is the most effective and sustainable treatment for obesity^3^. Roux-en-Y gastric bypass (RYGB), sleeve gastrectomy (SG), adjustable gastric banding and the biliopancreatic diversion, in combination with cognitive therapy and internal medicine, are the most successful treatment options for morbid obesity^4^. Endoscopic methods are recently evolving as a promising alternative for these patients. The endoscopically placed gastric balloon seems to be a safe transitional procedure for losing weight. The Apollo Overstich(r) is an endoscopic suturing device which has been currently used to perform gastric volume reduction in the style of the sleeve gastrectomy without resection of the stomach^5^. Another recent surgical procedure is the laparoscopic greater gastric curvature plication (LGCP). It is a procedure that reduces the gastric volume using sutures that invaginates the greater curvature, avoiding stapling or gastric resection, and aiming to reduce morbidity and costs for obesity surgery^6^ ^,^ ^7^. Although with good excess weight loss in clinical series, it still causes perioperative morbidity due to the gastric devascularisation without gastric resection. 

Our group recently presented a new technique for intraluminal gastric surgery using an intragastric single port device, the IGS-IGP technique, for a series of clinical application for resection of intragastric benign tumors^8^. Adapting this access for IGS-IGP can potentially produce a new bariatric restrictive procedure with advantages over LGCP and the endoscopic sleeve gastroplasty. 

The objective of this study was to evaluate the effectiveness of IGP in promoting gastric volume reduction in an experimental set. 

## METHODS

**FIGURE 1:**
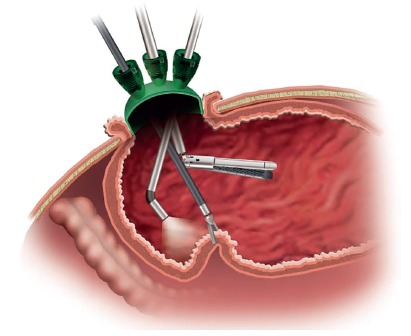
Schematic illustration for performing IGS-IGP for gastric volume reduction as a new restrictive bariatric procedure. A single port device is inserted in the antral part of the stomach, and variable laparoscopic instruments can be inserted for intragastric surgery

**FIGURE 2:**
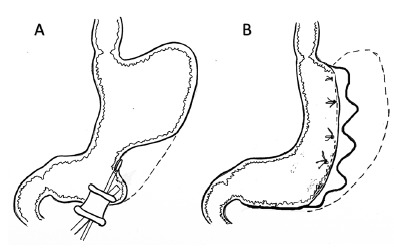
Schematic illustration for performing IGS-IGP for gastric volume reduction in an ex-vivo experimental swine model: A) a single port device is inserted in the antral part of the stomach, and laparoscopic intragastric suturing is performed using four interrupted sutures; B) intragastric greater curvature plication is started from the fundus region, progressing with further non-absorbable sutures to the antrum and, finally, the entry incision is closed with running suture

**FIGURE 3:**
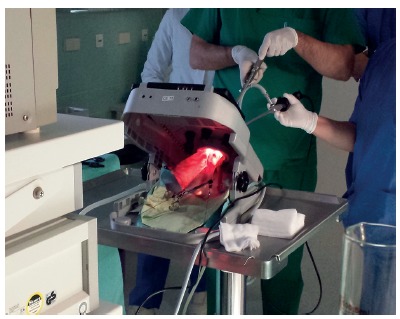
External view and team position at laparoscopic trainer for single port intragastric surgery (IGS) for internal sleeve plication

Between November and December 2015, 20 procedures were performed in the experimental surgery at our institution. The experimental model consisted of ex-vivo fresh pig cadaver stomachs installed in a laparoscopic single port trainer (Applied Medical, Rancho Santa Margarida, CA, USA) (Figures 1-3). 

**FIGURE 4:**
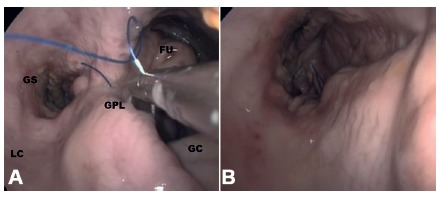
A) Internal laparoscopic view after the first suture performed at the gastric fundus (FU) and the resulting gastric sleeve tubular anatomy (GS) is shown, as the gastric suture plication (GPL) is performed along the greater curvature (GC), leaving the lesser curvature (LC) intact; B) final aspect of gastric reduction and altered anatomy by intragastric greater curvature plication

**FIGURE 5:**
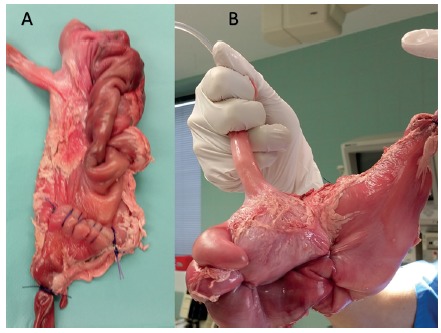
A) Final external aspect after concluding IGS internal sleeve plication; B) measuring the gastric volume after the anatomical modification, filling the stomach with high pressured (50 mmHg) of water

**FIGURE 6:**
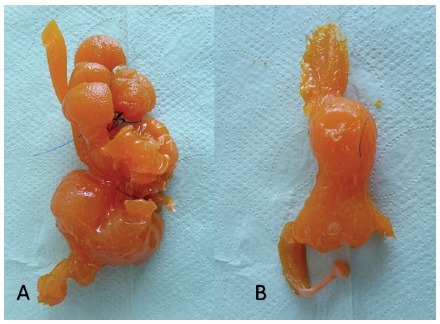
Plastinate model with cyanoacrylate showing differences in postoperative anatomy after IGS-IGP (A) and sleeve gastrectomy (B)

The procedure was performed as follows in ten pigs: 1) for measuring the initial pre-procedural gastric volume, each stomach was filled with water to a pressure of 50 mmHG (0.06 Bar); 2) the stomach wall was then extroverted and fixed to the laparoscopic trainer single port orifice, and opened a 2 cm anterior incision close to the pylorus; 3) the single port device (Gelpoint Applied Mini, Applied Medical, Rancho Santa Margarida, CA, USA) was inserted intragastrically through the 2 cm gastrotomy (Figure 2 A-B); 4) the lumen was insufflated inside the simulator with CO2 (1-2 mmHg), and a 30 degrees optic, a laparoscopic needle holder and a grasper were inserted through the single port; 5) four handsewing full-thickness 4-point sutures with Prolene 2-0 were performed from the fundus to the antrum in each stomach (Figure 4 A-B); 6) after finishing the procedure, the entrance defect was closed with Vicryl-0 running suture and the stomach was filled with water under pressure again and the residual volume was measured (Figure 5). 

In further ten pigs, sleeve gastrectomy was performed by stapling the antrum and greater curvature over a 36F bougie. Post-procedural gastric volume was also measured. As a result of the internal plication, the technique produced macroscopic alterations in the form and capacity of the plicated stomach (Figure 5). Plastinate models were produced filling the alterated specimens with cyanoacrylate to evaluate internal luminal anatomy for IGP and SG (Figure 6). A summary of the procedure can be reviewed in the video (video1).

## RESULTS

A total of 10 internal gastric plication procedures and 10 sleeve gastrectomy procedures were performed in an ex-vivo swine cadaver stomach model. Mean total procedure time for the plications was 27±4 min per stomach. The mean preoperative volume of the pig stomachs was 884 ml (510ml-1260ml). The mean volume after the plications was 341±169 ml. The mean volume after the sleeve gastrectomy was 63±23ml. The mean percentage of the volume reduction for IGS was 51±25% (Table 1). The mean volume reduction after sleeve gastrectomy was 90±5%. There was a high significantly difference between postoperative gastric volume, after IGP and after SG (p<0.005). 

**TABLE 1:** Intragastric water volume measure of specimens before surgery and after IGS-IGP

Pig	Volume preoperative in ml	Volume postoperative in ml	Volume reduction in %
1	800	740	8
2	610	420	31
3	510	440	14
4	1000	350	65
5	1000	340	66
6	710	240	66
7	510	200	61
8	510	250	51
9	900	290	68
10	740	140	81
	Mean 683 ml	Mean 341 ml	Mean 51%

Plastinate models were produced filling the lumen of procedural alterated specimens with colored cyanoacrylate to evaluate internal luminal anatomy for IGP and SG. The models showed the altered anatomy after the plication and the disposition of the remaining cavities (Figure 6).

## DISCUSSION

Bariatric surgery is the most effective and sustainable treatment for obesity^3^. When compared with each other, certain procedures resulted in greater weight loss and improvements in comorbidities than others. Outcomes were similar between RYGB and SG, and both of these procedures had better outcomes than adjustable gastric banding. Although with optimal results in reducing weight and ameliorating co-morbidities for these patients, RYGB and SG are associated with some complications due to the technical issues of stapling and anastomosing, with a risk of leaks and fistulas reported in nearly 1% of the cases^9^. 

New less invasive methods have been developed to promote an effective therapy without the potential problems of penetrating or resecting the gastric wall. To reduce the capacity of the stomach and allowing mechanical restriction to food intake is a known effective mechanism to promote weight loss for morbidly obese patients^9^. At least three current bariatric and metabolic procedures are based on gastric volume reduction, as the laparoscopic adjustable gastric banding (LAGB), SG and laparoscopic greater curvature plication (LGCP). 

O'Brien et al^10^ described a 15 year follow up for patients with adjustable gastric banding. They included 3227 patients, with a mean body mass index of 43,8 kg/m^2^, and 714 had completed at least ten years of follow-up. They showed 47% of excess weight loss at 15 years. Angrisani et al compared the laparoscopic adjustable gastric banding with the RYGB with 51 patients in a period of five years^11^. Mean BMI before surgery was 43 kg/m^2^. The mean BMI at 12 months after surgery was 35 for the RYGB and 39 for the LAGB, decreasing to 29 for the RYGB and 36 for the LAGB after three years and staying the same for the RYGB at 29 and still decreasing for the LAGB at 35 after five years. Excess weight loss was significantly higher in the RYGB group after five years (67% RYGB and 48% LAGB). Himpens et al^12^ showed an excess weight loss of 73% three years after and 58% six years after SG, similar to the findings of Diamantis et al^13^, with an EWL of 62% at five years after and 54% at six years after SG. 

Talebpour et al shared their 12 years experience in 800 patients submitted to LGCP^14^. The mean BMI of their patients was 42 kg/m^2^; the mean EWL was 20% after one month (n=779), 67% after 12 months (n=491), 70% after 24 months (n=356), 66% after three years (n=251), 62% after four years (n=176) and 55% after five years (n=134) following surgery. Severe complications occurred in 1% of the patients, including gastric necrosis and enteric fistulae. They had to perform operative revisions because of regain in 32 cases and failure in six cases. These findings show in the literature similar effects in EWL of LGCP to the SG^13^ ^,^ ^15^. In a systematic review, the complication rate of the LGCP seems to be higher^16^. There were minor complications with a rate of 11%, such as nausea and vomiting and major complications including bleeding, leaks, gastric obstruction and gastric fistula in 4%. Still, the procedure has not gained much popularity due to the possibility of these complications, compared to the low rates in the literature for RYGB or SG.

Endoscopic alternatives were developed to fill the gap between bariatric procedures with high effectiveness but with more complications, and the conservative therapy, less harmful but less efficient in obtaining sustainable weight loss. The endoscopically placed gastric balloon seems to be a safe procedure for losing weight, but a metanalysis showed that it is only a short-term effective treatment, but it is not yet capable to maintain a weight loss over a long period of time^17^. However, this therapy only seems to be successful in very compliant patients and weight regain after removal of the balloon is common^17^ ^-^ ^20^. 

The novel endoscopic suturing device available in the market, Apollo Overstich(r), has been currently used to perform a gastric volume reduction in the style of the SG without resection of the stomach^5^. Abu Dayyeh et al used this device in 2013 in four patients with a mean BMI of 36 kg/m^2^. The procedure time was 172 to 245 min and they placed 26 sutures^5^. Lopez-Nava et al used the Apollo overstitch as well in 50 patients^21^. The mean procedure time was 66 min and they placed seven sutures on average with five stitches in each suture. They did not measure the volume of the stomach pre- and post-intervention. Oral contrast studies in addition with an endoscopy were optional for the patients at 24 h, three and six months. The mean BMI changed from 37.7±4,6 kg/m^2^ to 30.9±5,1 kg/m^2^ at one year follow up. The indication of this therapy is currently applied to low-BMI patients^5^ ^,^ ^21^ ^,^ ^22^, but indication for superobese as a first step procedure for a 2^nd^ stage strategy is recently described^23^ ^,^ ^24^. The technique seems promising, but long-term results and studies with larger series are needed to evaluate the effectiveness of this method in the morbidly obese population. 

The physiology of our proposed IGP technique is potentially similar to the endoscopic gastric plication and not similar to the laparoscopic gastric plication, where the greater curvature is denervated and devascularized, and the plicated stomach wall is compressed to the lumen. In IGP, there is rather a delaying in gastric emptying caused by the shortening of the stomach and the impaired motility due to the sutures. In fact, in a recent study evaluating postoperative scintigraphic findings on patients submitted to endoscopic sleeve gastroplasty, Abu Dayyeh reported a delayed gastric emptying, recognizing a complete different mechanism of action of this therapy, if compared with SG, where on the contrary a rapid gastric emptying is expected^25^. 

The results of our study show that the IGP-IGS technique is feasible and produces, ex-vivo, a significant reduction in the gastric volume by restricting its area of distension. It is possible to reduce the volume of the stomach without resecting or bypassing it. It is also possible to perform a surgical gastric plication without devascularization, by acting intraluminally. The entry wound in the antral region does not produces relevant morbidity in our clinical experience with IGS for resection of intragastric tumors. In studies, LGCP seems to be as effective as the sleeve gastrectomy regarding EWL and ameliorating comorbidities. The IGP-IGS can potentially be as effective as that with less operative time, as there is no need to entry the rest of the peritoneal cavity nor to liberate the gastric vessels or vessel ligation. Leaks, gastric necrosis or bleeding are improbable, as the technique does not induce gastric ischemia. A shorter learning curve is expected, it is a procedure with only one incision and only one technical step. It also is reversible by just cutting the suture lines, endoscopically, within the first weeks after the operation. As the endoscopic similar technique - endoscopic sleeve gastroplasty - it can be followed by surgical malabsorptive methods as a second stage operation in cases with insufficient weight loss without any change to previous surgery.

Survival studies have to be addressed to evaluate the feasibility and safety of the technique. 

## CONCLUSION

The consequences of this study cannot be applied yet in the clinical practice, as experimental survival studies are needed to prove the feasibility and safety of IGP. Its evaluation as a valuable method for therapy of morbid obesity, in achieving sustainable weight loss, is the next step of our research.
